# Aging and rare diseases: from epidemiology to a call to action

**DOI:** 10.1007/s41999-025-01351-4

**Published:** 2026-02-23

**Authors:** Monica Mazzucato, Giulia Fanton, Andrea Vianello, Cinzia Minichiello, Laura Visonà Dalla Pozza, Ema Toto, Laura Pastori, Chiara Ceolin, Marina De Rui, Alessandra Coin, Giorgio Perilongo, Giuseppe Sergi

**Affiliations:** 1 Rare Diseases Coordinating Centre, Padua University Hospital, Veneto Region, Padua, Italy; 2 Department of Child and Maternal Health, Padua University, Padua, Italy; 3 Geriatrics Division, Department of Medicine (DIMED), Padua University Hospital, Padua, Italy

**Keywords:** Aging, Rare disease, Registry, Care transition, ORPHAcodes, Epidemiology

## Abstract

**Aim:**

Aging of the general population, advances in diagnostic and therapeutic strategies, and care improvements are making rare diseases (RDs) an emerging phenomenon in the geriatric population. This study aims to provide a snapshot of the growing population of older RD patients using population-based data from the Veneto region rare disease registry (VRRDR), Italy.

**Methods:**

We report the number of patients diagnosed in old age and the number of those transitioned from adulthood into old age, estimating the prevalence of older RD patients as of 31 December 2022. Finally, we analyzed the composition of their therapeutic plans.

**Results:**

During the study period, 8975 patients received an RD diagnosis after 65 years of age, while 4214 patients diagnosed in their childhood or adulthood experienced the transition to old age. On 31 December 2022, there were 9508 patients aged 65 years or more residing in the study area, 20.8% of all Veneto’s RD patients. The most common groups of RDs in older patients are systemic or rheumatologic, neurologic, and skin diseases, affecting 27%, 25%, and 9% of the monitored geriatric population, respectively. Among prevalent cases, 1519 patients older than 65 years had a therapeutic plan related to their rare condition. Specifically, the most prescribed drugs were those affecting the nervous system (27.8%), the alimentary tract and metabolism (12.8%), and the antineoplastic and immuno-modulating agents (11.6%).

**Conclusions:**

As older patients represent a growing number in the RD population, geriatricians and general practitioners should be involved in RD care and training programs. Further studies are needed to determine the unmet care needs of older RD patients in order to design health policies able to address the peculiar challenges posed by this group of patients emerging within the RD population.

**Supplementary Information:**

The online version contains supplementary material available at 10.1007/s41999-025-01351-4.

## Key summary points

**Aim:**

To investigate the epidemiology of older people living with a rare disease (RD) using data from a population-based RD registry ongoing in the Veneto region of Italy, referring to the period 2002–2022.

**Findings:**

Patients aged over 65 years represent a significant proportion of all patients living with an RD in the monitored area at the end of the study period (20.8%). The most represented disease groups in the geriatric RD population are neurologic (36.7%), systemic or rheumatologic (25.1%), and skin diseases (11.3%).

**Message:**

The number of older people living with a rare disease (RD) is increasing, posing several challenges for both patients and healthcare providers, who need to be aware of the peculiarities of this twice-as-frail population.

**Supplementary Information:**

The online version contains supplementary material available at 10.1007/s41999-025-01351-4.

## Background

A rare disease (RD) is a complex health condition that affects a few people in the general population, with a cut-off threshold varying by country. In Europe, the prevalence should not exceed five cases per 10,000 individuals [1]. According to recent estimates, the RD cumulative prevalence ranges from 3.5 to 5.9%, corresponding to 263 to 446 million people worldwide having one of the approximately 8000 known RDs [2]. Thus, although RDs are individually uncommon, their global burden is far from negligible, making these medical conditions a public health priority [3].

Commonly, RDs are perceived as a pediatric issue as nearly 70% of them have a reported onset in childhood, with the remaining 12% and 18% emerging in adulthood and at all ages, respectively [2]. Nonetheless, the majority of patients fall within the adult demographic, navigating specific needs in everyday life [3, 4]. The number of people receiving a diagnosis of an RD in their old age is increasing due to the aging of the general population and the increase in diagnostic abilities [5, 6]. At the same time, advancements in treatment options have elevated survival rates in conditions with unfavorable prognoses in the past, resulting in the transition of generations from adulthood into old age as observed in pediatric patients [7, 8]. In this rapidly evolving scenario, the RD landscape is undergoing epidemiological and social changes [9], and a new group of patients is emerging, facing the challenges of living with an RD in their old age [10, 11].

Some studies have provided insights into the epidemiology of RDs [2, 12, 13], but without focusing specifically on the older population.

This study aims to describe the epidemiology of aging in RD patients in the Veneto region of Italy. We report the number of patients diagnosed in old age and the number of those who transited from adulthood into old age, estimating the prevalence of older RD patients as of 31 December 2022. Finally, we described the drug use profile in RD geriatric patients, analyzing the composition of their therapeutic plans regarding the number and nature of treatments prescribed by RD centers of expertise.

## Methods

The present work is a registry-based retrospective cohort study. We used data from the Veneto region rare disease registry (VRRDR), ongoing since 2002. Veneto region is located in the northeast of Italy and has nearly 4.9 million inhabitants [14]. Based on the interaction between highly specialized hospitals and territorial services, the RD care network has been officially defined since 2002 and is monitored and periodically updated according to specific criteria. For the purpose of this study, we considered diseases included in the Italian list of RDs [15] and in the Orphanet nomenclature [16]. Patients affected by RDs indexed in the national list can benefit from an exemption from medical charges and other aids, such as drugs, visits, and medical devices. All medical prescriptions, designated by a Centre of expertise, define the patient’s therapeutic plan. The VRRDR, the RD care network, and the related activities have been previously described [7, 12].

The RD care network is organized on three levels:

1. Centers of expertise: labeled on the basis of activity data derived from independent sources, in charge of performing the diagnostic and care activities for RD patients;
2. Centers of expertise part of the European Reference Network (ERN) for RDs, namely “Centres of excellence”;
3. Coordinating Center: with the task of governing and monitoring the network and operating the RD registry.

The VRRDR is interoperable with the regional administrative health database (AHD), which includes demographic information and the residential history of all the potential healthcare beneficiaries living in the region. For identifying patients diagnosed in old age and those transited from adulthood into old age, we considered all patients born until 1957 (i.e., all patients potentially 65 years old or above on 31 December 2022) and meeting the inclusion criteria. For calculating prevalent cases, we considered the number of patients aged above the cut-off threshold of 65 years old and residing in the study area on 31 December 2022 (point-prevalence); prevalence was calculated for the total sample and per province of residence. Diagnoses are recorded in the registry using the ICD-9-CM and ICD-10, OMIM and ORPHAcodes [17]. The information about the preferential parent, as derived from the Orphanet nomenclature pack, was used to group RDs diagnoses [16]. In the case of a double diagnosis, we considered the more recent one for the analyses.

For the analysis of the therapeutic plans, we used the Anatomical Therapeutic Chemical (ATC) classification system for grouping prescribed drugs [18].

We conducted data management and analyses using SAS (version 9.4M6) and R software (version 4.2.1) through the RCommander graphical user interface (GUI), and the Microsoft Office suit and MapChart.net for graphs, tables, and figures.

The Veneto region RD registry, as the other Italian regional/interregional RD registries and the National RD Registry, is established and regulated by Law (Ministerial Decree 279/2001, DPCM 3.03.2017; Law n.175/2021). Patient consent was waived as the RD Registry is established by law and respects the national data protection regulations. No individual data have been considered in the study analysis.

## Results

**Fig. 1:**
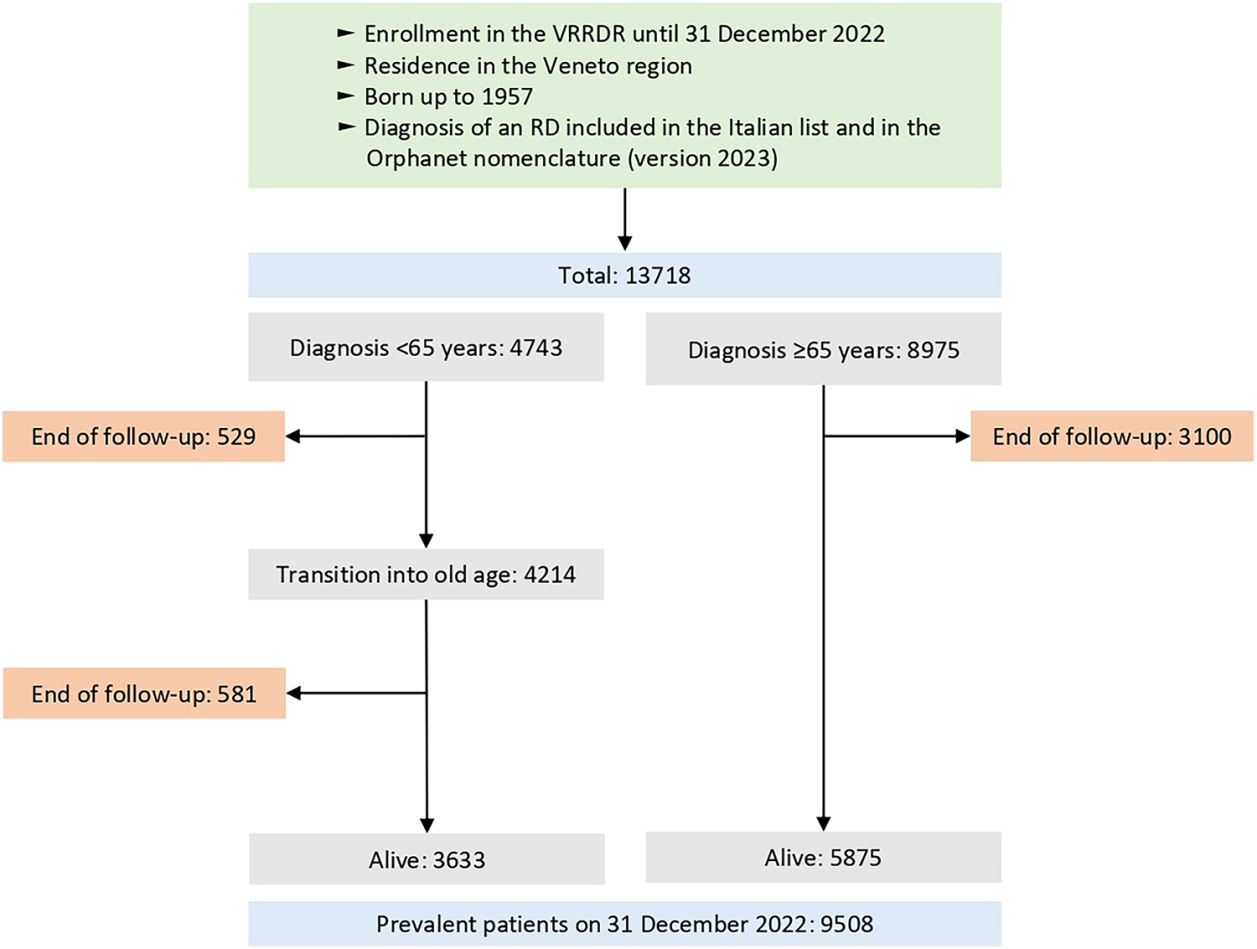
Flow diagram of the selection process of the patients included in the analyses

Each patient included in the RD regional registry was followed from the date of first diagnosis up to 31 December 2022, date of death or emigration outside the study area. As of 31 December 2022, 13,718 individuals (47.7% males) residing in the Veneto region and born until 1957 received an RD diagnosis performed by an expert center of the RD care network. 8975 patients were diagnosed in old age and 5875 of them were alive at the end of the study period. Conversely, 4743 patients received an RD diagnosis before the age of 65 years. Overall, 4214 patients experienced the transition into old age, and 9508 patients aged 65 years old or over were living with an RD on 31 December 2022 (see Fig. 1) in the study area. None of the patients considered in the present study presented multiple RD diagnosis. Patients' characteristics are reported in Table 1.

**Table 1:** Characteristics of all patients included in the study, overall and according to age of diagnosis

	Diagnosis:			
	< 65 years		≥ 65 years	
Patients, N (%)	**4743**	**(100)**	**8975**	**(100)**
Age (years) at diagnosis, mean ± SD	58.8	± 4.8	74.2	± 6.2
Age (years) at end of follow-up, mean ± SD	69.2	± 5.2	78.4	± 6.5
Female, N (%)	2448	(51.6)	4733	(52.7)
Orphanet classification, N (%)				
Rare neurologic diseases	1236	(26.1)	3293	(36.7)
Rare systemic or rheumatologic diseases	1006	(21.2)	2255	(25.1)
Rare skin diseases	336	(7.1)	1016	(11.3)
Rare hematologic diseases	439	(9.3)	530	(5.9)
Rare inborn errors of metabolism	518	(10.9)	230	(2.6)
Rare ophthalmic disorders	321	(6.8)	423	(4.7)
Rare respiratory diseases	60	(1.3)	364	(4.1)
Rare gastroenterological diseases	206	(4.3)	211	(2.3)
Rare developmental defects during embryogenesis	187	(3.9)	153	(1.7)
Rare neoplastic diseases	190	(4.0)	91	(1.0)
Rare immune diseases	51	(1.1)	159	(1.8)
Rare urogenital diseases	85	(1.8)	111	(1.2)
Rare endocrine diseases	50	(1.0)	85	(1.0)
Other RDs	58	(1.2)	54	(0.6)

Abbreviations: *SD* standard deviation, *RD* rare disease

### Patients diagnosed in old age

A total of 8975 patients (47.3% males, mean age of 74.2 years) received a diagnosis of an RD after reaching the age of 65 years. Among them, 3076 (34.3%) died during the study period, with an average age at the time of death being 79.3 years (SD 7.1), and 24 moved outside the study area. Most of the patients (94.4%) received their diagnosis from RD centers located in the Veneto region. Characteristics are shown in Table 1.

The most represented disease groups in the geriatric RD population are neurologic (36.7%), systemic or rheumatologic (25.1%), and skin diseases (11.3%). In detail, the most common diagnoses are myasthenia gravis (976 cases, 10.9%), systemic sclerosis (939 cases, 10.5%), amyotrophic lateral sclerosis (ALS, 899 cases, 10%) and bullous pemphigoid (658 cases, 7.3%).

### Patients transited from adulthood to old age

**Fig. 2:**
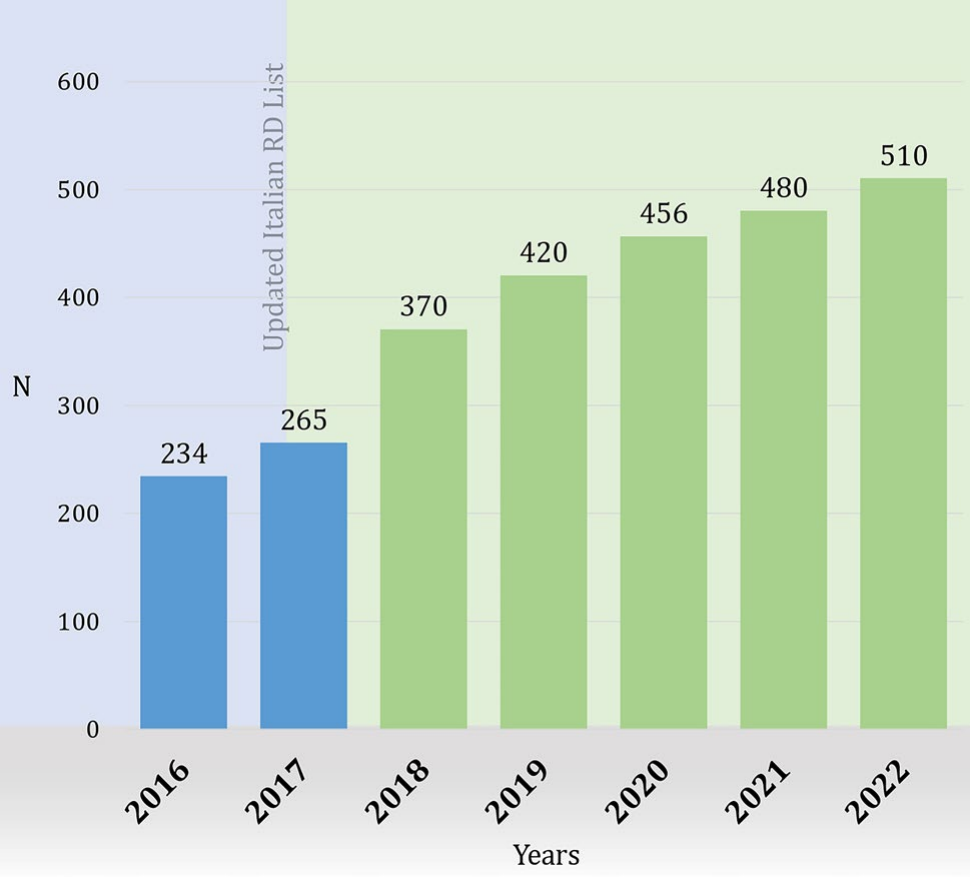
Older RD patients experiencing the transition from adulthood to old age (2016–2022)

During the study period, 4743 patients (48.4% males) born up to 1957 were diagnosed before the age of 65, at a mean age of 58.8 years (SD 4.8). Approximately 4214 patients (46.9% males) reached the age of 65 years; patients experiencing transition accounted for 31.9% of all older patients registered throughout the years (Table 2). As shown in Fig. 2, over the years there has been an increase, in absolute terms, of RD patients reaching the age of 65. Among the 10 most prevalent RDs diagnosed in patients reaching old age, the majority have an onset primarily during adulthood (i.e., rare hereditary hemochromatosis, systemic sclerosis, and ALS; see Online Resource 1).

**Table 2:** Characteristics of RD patients experiencing the transition from adulthood to old age

Patients, N (%)	**4214**	**(100)**
Age (years) at diagnosis, mean ± SD	59.1	± 4.8
Age (years) at end of follow-up, mean ± SD	70.3	± 4.3
Patients diagnosed by a RD Center in Veneto region, N (%)	3381	(94.5)
Orphanet classification, N (%)		
Rare systemic or rheumatologic diseases	940	(22.3)
Rare neurologic diseases	904	(21.5)
Rare inborn errors of metabolism	488	(11.6)
Rare hematologic diseases	413	(9.8)
Rare skin diseases	319	(7.6)
Rare ophthalmic disorders	309	(7.3)
Rare developmental defects during embryogenesis	180	(4.3)
Rare gastroenterologic diseases	179	(4.2)
Other RDs	482	(11.4)

Abbreviations: *SD* standard deviation, *RD* rare disease

### Prevalence

As of 31 December 2022, there were 9508 (45.7% males) patients aged 65 years or more residing in the study area, representing the 20.8% of all the RD patients monitored by the registry.

Among those, 3633 participants (38.2%) were diagnosed before the age of 65, thus experiencing the transition from adulthood to old age. 8987 (94.5%) patients received a diagnosis in an RD center located in the Veneto region, and 521 (5.2%) in RD centers located outside the region. The prevalence rates differ between provinces (Online Resource 2).

**Fig. 3:**
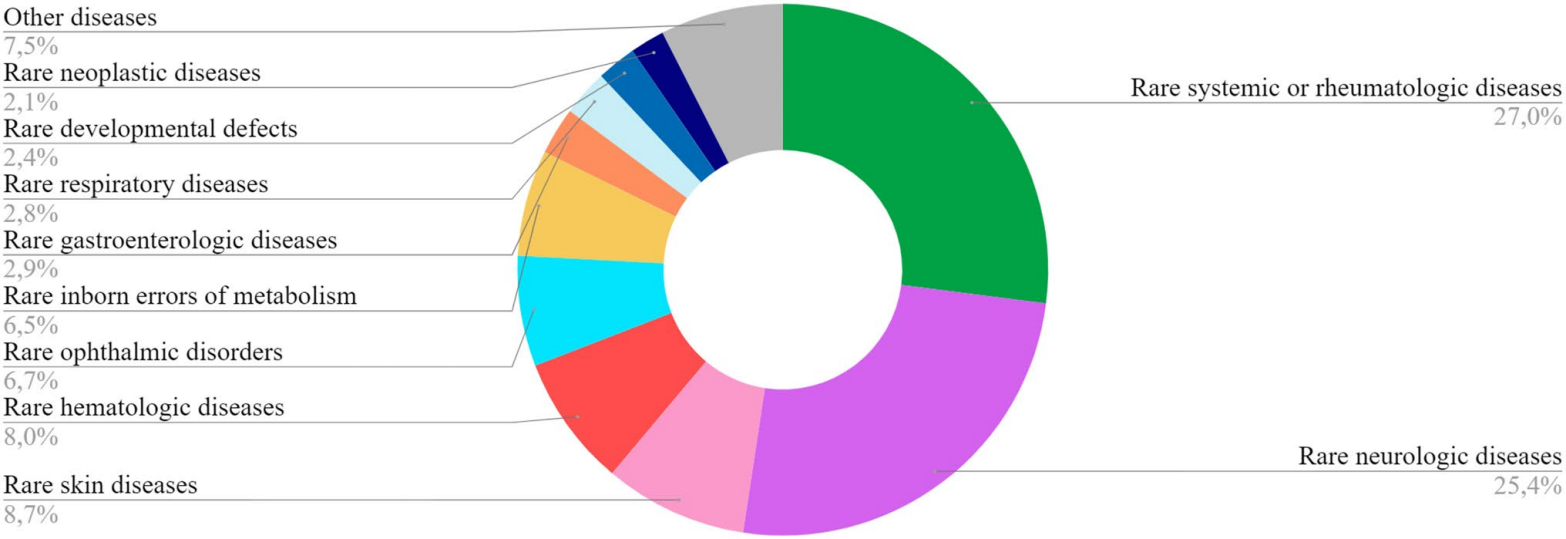
Distribution of RDs in older patients (≥ 65 years) by Orphanet classification on 31.12.2022

The most prevalent groups of RDs in the geriatric population under study are systemic or rheumatologic, neurological, and skin diseases (Fig. 3). In detail, RD with the highest incidence in the geriatric age group are: systemic sclerosis (994 cases, 10%), myasthenia gravis (914 cases, 9.6%), rare hereditary hemochromatosis (477, 5%), giant cell arteritis (339 cases, 3.6%), bullous pemphigoid (334 cases, 3.5%), chronic inflammatory demyelinating polyneuropathy (297 cases, 3.1%), retinitis pigmentosa (237 cases, 2.5%), idiopathic pulmonary fibrosis (228 cases, 2.4%) and idiopathic achalasia (213 cases, 2.2%).

### Treatment prescription profile

Among 9508 prevalent older patients, 1519 had a therapeutic plan related to their RD issued in 2022.

The total number of prescribed products amounted to 3117, mainly drugs (66.9%; see Table 3). Consistent with the RDs most commonly represented in the monitored geriatric population, drugs affecting the nervous system made up nearly 28% of all prescribed drugs. For example, the analysis of drug prescriptions for the treatment of ALS patients (*n* = 174) showed a combination of riluzole, having an RD-specific indication, and symptomatic treatments, accounting for *n* = 114 (65.5%) and *n* = 60 (34.5%) of all prescriptions, respectively.

**Table 3:** Composition of the therapeutic plans of prevalent older patients (≥ 65 years) on 31.12.2022

	N	**%**
Products	**3117**	**100**
Drugs	2084	66.9
Medical Foods	537	17.3
Medical Devices	286	9.2
Prosthetics	136	4.4
Galenic preparations	74	2.4
Drugs: ATC classification	**2084**	**100**
Nervous system	581	27.9
Alimentary tract and metabolism	268	12.9
Antineoplastic and immunomodulating agents	243	11.7
Blood and blood-forming organs	214	10.3
Anti-infective for systemic use	211	10.2
Cardiovascular system	155	7.5
Systemic hormonal preparations^a^	104	5.0
Dermatologicals	98	4.7
Sensory organs	62	3.0
Others	148	7.1

^a^Excluding sex hormones and insulins. Abbreviations: *ATC* anatomical therapeutic chemical

## Discussion

Our study highlights that a considerable number of patients receive a RD diagnosis after the age of 65, with a significant portion transitioning from childhood or adulthood to old age. To our knowledge, this is the first attempt to provide epidemiologic and real-world drug prescription figures referred to older patients living with an RD using data from a population-based registry.

Since the beginning of the VRRDR activity, 8975 patients were diagnosed when they were 65 years or older, and 4214 experienced the transition from adulthood to old age living with an RD. According to our findings, patients aged over 65 years represent a significant proportion of all patients living with an RD in the monitored area at the end of the study period (20.8%). These findings are not directly comparable with other studies investigating the epidemiology and the healthcare utilization in patients diagnosed with RD as those data usually refer to two distinct age population subgroups, pediatric and non-pediatric, or are based on hospital-based data collection [19–22]. In general, data referring to older age classes are seldom available, with studies usually focusing on a limited number of RD [23]. A Chinese study analyzed the medical records of 78 tertiary and community hospitals providing data on the economic burden of 23 RDs. Patients older than 65 years were 10.9% of all the identified RD patients [24].

RDs represent a highly heterogeneous group comprising diseases with different onset age and trajectories that vary widely depending not only on the type of condition, as well as patients’ age and sex [13, 25–27]. For example, systemic sclerosis, which in the registry accounts for more than 10% of the diagnoses made after the age of 65 years, predominantly affects women, who can present with different clinical manifestations than men [28, 29]. Regarding myasthenia gravis, another main RD in the present study population, recent studies document a bimodal incidence pattern, with a first peak around the fourth decade of life in women and a second one after the sixth decade, a finding that was also observed in our cohort (Online Resource 3) [30, 31]. Importantly, the challenges faced by patients aging with a pediatric-onset disease, such as an inborn error of metabolism, differ substantially from those encountered by older patients who receive a late diagnosis of a progressive neurodegenerative disease [32, 33]. The majority of patients in the VRRDR who reached the age of 65 had an adult-onset rare disease, as in the case of ALS, while a small subgroup was affected by RDs with onset in childhood or adolescence, such as hemophilia A. Future research in the geriatric population focusing on specific diseases should consider these specific aspects to better capture differences in clinical trajectories, functional outcomes and care needs and to inform tailored management strategies.

As regards the disease groups, in our study, more than half of the RD geriatric patients who were still alive by the study's conclusion had a rare systemic or rheumatic disease or a rare neurological condition. Moreover, 40% of cases could be attributed to a limited number of RD entities, with systemic sclerosis and myasthenia gravis each accounting for 10% individually. This is in accordance with previous epidemiological studies on these two RDs showing higher prevalence values in older individuals and onset age progressively shifted to the old age [34–41]. Consistent with the Pareto principle, data presented confirm that, despite the high number of monitored RDs, only a limited proportion of them constitute the majority of cases. These findings may have important implications in designing care pathways and tailored training programs for health professionals [42]. According to the results from a national survey carried out in Italy in 2020, geriatrics had limited knowledge of RDs, even in the case of a condition such as cardiac amyloidosis, which typically affects older patients [43]. This can lead to misdiagnosis, and delay in diagnosis and treatment initiation [44].

Besides focusing on the changing demographic profile of the RD population over the years, we were further interested in describing the characteristics of the therapeutic plans of geriatric RD patients. In our study, only a limited proportion (16%) of the prevalent patients had a therapeutic plan active at the end of 2022. This finding may be related to the fact that we considered mainly treatments prescribed for RDs, rather than reflecting an under treatment phenomenon. Data on supportive treatments might have been underestimated, especially when not directly linked to the patient's RD status. In a previous study carried out on the same population, we found that orphan medicinal product (OMP) prescriptions involved only a limited proportion of RD patients (2.3%) [45]. Although mandatory prescriptive forms and specific sections for high-cost or innovative drugs have been included in the VRRDR to collect clinical information on specific treatment options, we cannot exclude the possibility that, in some cases, prescriptions for patients who did not require specific RD drugs may not have been recorded in the registry. Therefore, further studies are needed in the monitored population to investigate the real impact of RD-related treatments versus treatments for common comorbidities, also considering that polypharmacy can lead to peculiar consequences [46–48].

The present study is affected by the following limits: first, possible cases’ underestimation. During its initial years of activity, the registry had not yet reached full capacity, so corresponding data may not precisely reflect the true extent of the aging phenomenon during that time. Additionally, Walter et al. [49] showed a higher risk of being underdiagnosed or misdiagnosed for individuals with an RD living in more remote residential areas. This can apply also to our population, translating into an increased probability that older patients living far from RD centers can be underrepresented. The impact of telemedicine in reducing the geographical barriers and expanding access to specialized care in geriatric RD patients should be further investigated [50]. Nevertheless, we think that the possible initial underestimation has been progressively overcome as enrollment in the RD registry is the prerequisite for exemption from healthcare costs related to the RD diagnosis and is monitored by the Regional Health Authority to confirm the labeling of the Centers as RD expert ones. Therefore, it is conceivable that the RD registry has reached a good population coverage over time.

A second limitation is that the list of monitored RDs, although it includes a broad range of entities and has been expanded over the years, does not fully cover all the RDs represented in the Orphanet nomenclature. To overcome this limit, the VRRDR has introduced the possibility of registering other RDs included in the Orphanet nomenclature, but not present in the Italian official RD list yet. Nevertheless, as this additional registering activity is not mandatory, the population coverage for these diseases may be reduced, and this is the reason why data referred to these RDs have not been considered in the present study.

In addition, given the main epidemiological nature of the VRRDR, we did not capture validated clinical assessments of comorbidity, functional status, cognition, or nutritional status. Therefore, frailty was not directly assessed. According to the findings of the present study, the incorporation of a frailty index for older patients represents an aspect of future development of the Registry. By adopting a multi domain frailty assessment based on indexes as the multidimensional prognostic index (MPI), future studies could move from inference based on epidemiological proxies to a direct, standardized quantification of frailty [51].

On the other hand, the main strengths of the present study are the long monitoring period and the size of the population under study, including patients of all ages. Furthermore, the adopted population-based approach prevents bias that can affect findings when based on data from disease-specific registries or derived from centers caring for selected groups of patients. Finally, our findings reflect the characteristics of the population and healthcare system organization in place in the study area. These results offer valuable insights, but caution is needed regarding their generalization to other populations as a specific geographical distribution exists, especially for some RDs [52–57]. Comparative analysis should take into account contextual differences in demographics, healthcare infrastructure, and population health profiles.

## Conclusions

To our knowledge, this is the first study describing the epidemiology of a consistent group of RD in older patients. Our results highlight an aging trend among RD patients, revealing a notable shift in the demographic profile of this population over the years. This emerging phenomenon poses several challenges for both patients and healthcare providers, who need to be aware of the peculiarities of this twice-as-frail population. Data provided by the present study can inform health services planning, highlighting not only the importance of identifying highly specialized centers, but also of establishing connections between them and the local services, both hospital and territorial ones. In this scenario, the role of general practitioners and geriatricians should be better acknowledged as they can represent the first and often the main point of access to care. The findings of this study, supporting a shift from a mere pediatric-centric view of these disorders to a broader perspective including the geriatric age, can have implications for the shaping of adapted healthcare policies and resource allocation. Further studies are needed to determine the unmet needs of RD older patients to elaborate adequate health policies, including the use of telemedicine, accompanying carefully RD patients progressively becoming older in their care trajectories.

## Supplementary Information

Below is the link to the electronic supplementary material.

Supplementary file1 (PDF 185 KB)

Supplementary file2 (PDF 329 KB)

Supplementary file3 (PDF 215 KB)

## Data Availability

The data that support the findings of this study are available from the corresponding author [MM] upon reasonable request and with permission of the Veneto region.

## Abbreviations

RD: Rare disease
VRRDR: Veneto region rare disease registry
EU: European Union
WHO: World Health Organization
UN: United Nations
SIGG: Italian Society of Gerontology and Geriatrics
ERN: European Reference Network
ATC: Anatomical therapeutic chemical
AHD: Administrative health database
GUI: Graphical user interface
SD: Standard deviation
CI: Confidence interval
ALS: Amyotrophic lateral sclerosis
