# Supplementary material for: Aging and rare diseases: from epidemiology to a call to action
Source: Eur Geriatr Med. 2026 Feb 23;17(2):1009–18. doi: 10.1007/s41999-025-01351-4 (PMC13109167; doi:10.1007/s41999-025-01351-4)
Supplement: Supplementary file 1 — Supplementary file1 (PDF 185 KB) [file 41999_2025_1351_MOESM1_ESM.pdf]

Title: Aging and rare diseases: from epidemiology to a call to action

Journal: European Geriatric Medicine

Authors

Monica Mazzucato[1,2], Giulia Fanton[1], Andrea Vianello[2], Cinzia Minichiello[1,2], Laura Visonà Dalla Pozza[1,2], Ema Toto[1,2], Laura Pastori[1,2], Chiara Ceolin[3], Marina De Rui[3], Alessandra Coin[3], Giorgio Perilongo[1,2], Giuseppe Sergi[3]

Institutional address

[1] Rare Diseases Coordinating Centre, Padua University Hospital, Veneto Region, Padua, Italy

[2] Department of Child and Maternal Health, Padua University Hospital, Padua, Italy

[3] Department of Medicine (DIMED), Geriatrics Division, Padua University Hospital, Padua, Italy

* Corresponding author: Monica Mazzucato monica.mazzucato@unipd.it; monica.mazzucato@regione.veneto.it

First 10 rare diseases (RDs) diagnosed in patients who experienced the transition from adulthood to old age

**RDs**	**N**	**%**
Rare hereditary hemochromatosis	377	8.9
Systemic sclerosis	218	5.2
Amyotrophic lateral sclerosis	211	5.0
Retinitis pigmentosa	147	3.5
Idiopathic achalasia	137	3.3
Myasthenia gravis	131	3.1
Chronic inflammatory demyelinating polyneuropathy	116	2.8
Mixed connective tissue disease	98	2.3
Eosinophilic granulomatosis with polyangiitis	77	1.8
Rare lichen planus	75	1.8
Others RDs	2627	62.3
Total RD diagnosis	4214	100
