# Supplementary material for: Aging and rare diseases: from epidemiology to a call to action
Source: Eur Geriatr Med. 2026 Feb 23;17(2):1009–18. doi: 10.1007/s41999-025-01351-4 (PMC13109167; doi:10.1007/s41999-025-01351-4)
Supplement: Supplementary file 2 — Supplementary file2 (PDF 329 KB) [file 41999_2025_1351_MOESM2_ESM.pdf]

Title: Aging and rare diseases: from epidemiology to a call to action

Journal: European Geriatric Medicine

Authors

Monica Mazzucato[1,2], Giulia Fanton[1], Andrea Vianello[2], Cinzia Minichiello[1,2], Laura Visonà Dalla Pozza[1,2], Ema Toto[1,2], Laura Pastori[1,2], Chiara Ceolin[3], Marina De Rui[3], Alessandra Coin[3], Giorgio Perilongo[1,2], Giuseppe Sergi[3]

Institutional address

[1] Rare Diseases Coordinating Centre, Padua University Hospital, Veneto Region, Padua, Italy

[2] Department of Child and Maternal Health, Padua University Hospital, Padua, Italy

[3] Department of Medicine (DIMED), Geriatrics Division, Padua University Hospital, Padua, Italy

* Corresponding author: Monica Mazzucato monica.mazzucato@unipd.it; monica.mazzucato@regione.veneto.it

Prevalence map of older RD patients (≥ 65 years) residing in the Veneto region (per 10,000 inhabitants)

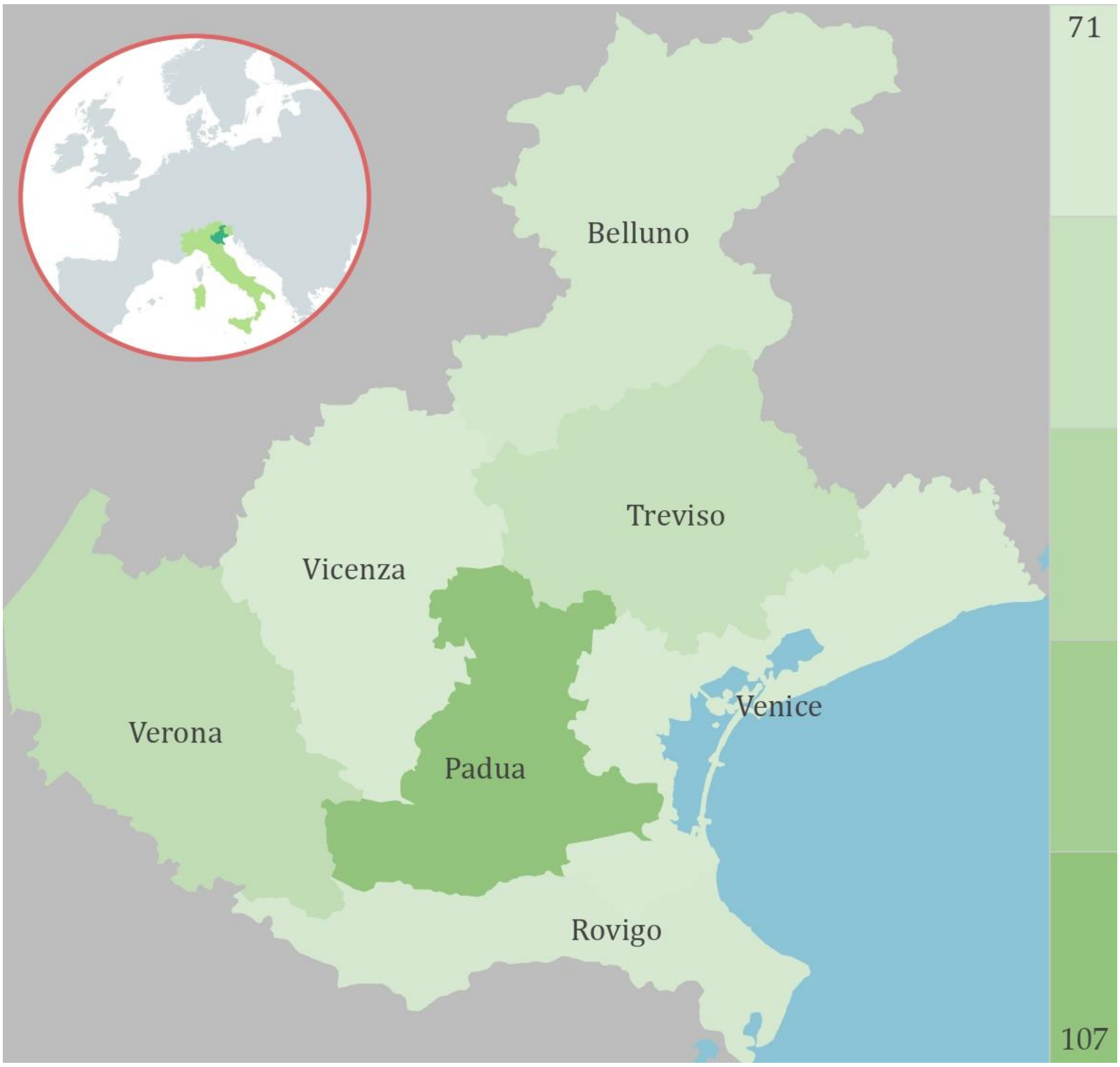

Prevalence rates of older RD patients (≥ 65 years) by Province of residence (Veneto region) on 31.12.2022

**Population ≥ 65 years old**	**Total**	**RD patients**	**Prevalence rate per 10,000**	**95% CI**
**Veneto region**	1167759	9508	81.4	79.8-83.1
**Provinces of Veneto**				
Verona	211880	1772	83.6	79.8-87.5
Vicenza	196713	1398	71.1	67.4-74.8
Belluno	54934	411	74.8	67.6-82.0
Treviso	205214	1565	76.3	72.5-80.0
Venice	214764	1534	71.4	67.9-75.0
Padua	221344	2368	107.0	102.7-111.3
Rovigo	62910	460	73.1	66.5-79.8

Abbreviations: *RD* rare disease, *CI* confidence interval. Data from I. Stat.
