# Supplementary material for: Aging and rare diseases: from epidemiology to a call to action
Source: Eur Geriatr Med. 2026 Feb 23;17(2):1009–18. doi: 10.1007/s41999-025-01351-4 (PMC13109167; doi:10.1007/s41999-025-01351-4)

**Title: Aging and rare diseases: from epidemiology to a call to action**

**Journal: European Geriatric Medicine**


**Authors**

Monica Mazzucato[1,2], Giulia Fanton[1], Andrea Vianello[2], Cinzia Minichiello[1,2], Laura Visonà Dalla Pozza[1,2], Ema Toto[1,2], Laura Pastori[1,2], Chiara Ceolin[3], Marina De Rui[3], Alessandra Coin[3], Giorgio Perilongo[1,2], Giuseppe Sergi[3]

**Institutional address**

[1] Rare Diseases Coordinating Centre, Padua University Hospital, Veneto Region, Padua, Italy

[2] Department of Child and Maternal Health, Padua University Hospital, Padua, Italy

[3] Department of Medicine (DIMED), Geriatrics Division, Padua University Hospital, Padua, Italy

* Corresponding author: Monica Mazzucato monica.mazzucato@unipd.it; monica.mazzucato@regione.veneto.it


**Age distribution of patients with myasthenia gravis at diagnosis (F: Female; M: Male)**

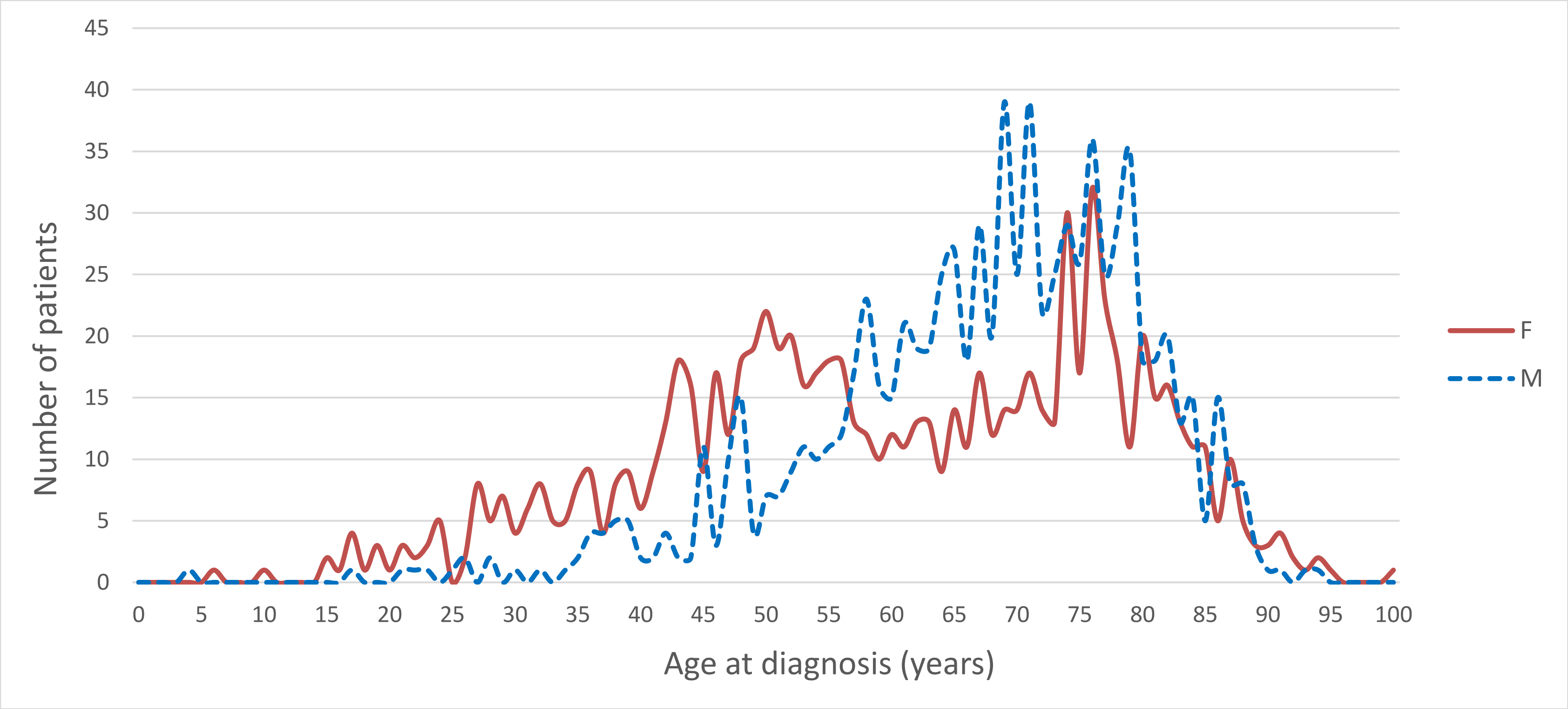

Supplement: Supplementary file 3 — Supplementary file3 (PDF 215 KB) [file 41999_2025_1351_MOESM3_ESM.pdf]
